# Headache symptoms of the PREEMPT population

**DOI:** 10.1186/1129-2377-14-S1-P99

**Published:** 2013-02-21

**Authors:** DW Dodick, RE DeGryse, CC Turkel

**Affiliations:** 1 Mayo Clinic Arizona, Dept. of Neurology, Phoenix, AZ, United States, USA; 2 Allergan, Irvine, CA, United States, USA

## Introduction

CM is a complex neurological disorder affecting approximately 2% of the general adult population. CM sufferers experience a broad range of debilitating symptoms.

## Objective

To assess the daily headache symptoms of chronic migraine (CM) patients over a 4-week period.

## Design/methods

PREEMPT (two phase 3 studies: 24-week, double-blind, placebo-controlled, parallel-group phase, followed by 32-week, open-label phase) evaluated onabotulinumtoxinA for prophylaxis of headaches in CM (>15 days/month with headache lasting >4 hours/day). A total of 1384 CM patients daily reported their headache symptoms for a 28-day baseline period using an interactive voice response telephone diary. The baseline frequencies of various headache features and associated symptoms were computed.

## Results

Of 38,752 total days, patients reported 27,483 (70.9%) days with >4 hours of headache. Patients classified their pain as moderate/severe on 90.9% of their headache-days. The most common associated symptoms patients described experiencing on headache-days were photophobia (81.2%), phonophobia (80.2%), exacerbation with physical activity (80.0%), pulsating quality (70.8%), unilateral pain (63.6%), and nausea (59.8%). Vomiting (13.8%) was reported infrequently.

## Conclusion/relevance

These CM patients experienced severe headache symptoms throughout the 28-day baseline period. The overwhelming majority of headaches were characterized as moderate/severe pain intensity, which were often accompanied with sensitivity to both light and sound and aggravated by routine physical activity. The majority of patients also reported pulsating pain quality, unilateral headache, and nausea. The heavy burden of illness suffered by CM patients emphasizes the necessity of prophylactic treatment for their headaches.

## Support

Allergan, Inc.

